# The Predictive Relationship Between Sensory Reactivity and Depressive Symptoms in Young Autistic Children with Few to No Words

**DOI:** 10.1007/s10803-022-05528-9

**Published:** 2022-03-26

**Authors:** Timothy Rossow, Keren MacLennan, Teresa Tavassoli

**Affiliations:** University of Reading, Harry Pitt Building, Earley Gate, Reading, RG6 6BZ UK

**Keywords:** Autism, Sensory reactivity, Depression, Children, Language delay

## Abstract

Depression and sensory reactivity are both common in autism. However, there is little understanding of the predictive relationship between these factors, or the nature of this relationship in autistic children who speak few to no words. This study set out to explore the longitudinal relationship between sensory reactivity and depressive symptoms in 33 young autistic children who speak few to no words. We found positive correlations between depressive symptoms and hyper-reactivity and sensory seeking at both timepoints, and across timepoints. We further found a bidirectional predictive relationship between depressive symptoms and sensory seeking. These results implicate sensory seeking in the development of depressive symptoms in young autistic children who use few to no words. Our findings have important implications for preventative mental health interventions, especially for those with a developmental language delay.

**Supplementary Information:**

The online version contains supplementary material available at 10.1007/s10803-022-05528-9.

There has been much interest in sensory reactivity since its inclusion as a diagnostic criterion for autism in the most recent edition of the Diagnostic and Statistical Manual of Mental Disorders (DSM-5) (American Psychiatric Association, 2013). Whilst several methods for classifying different sensory constructs exist (Dunn, 1997, 2001; Miller et al., 2007), the DSM-5 classifies sensory reactivity into three constructs dependent upon the way in which sensory stimuli are responded to—sensory hyper-reactivity (having a sensitivity to sensory stimuli, such as being overwhelmed by tactile input), sensory hypo-reactivity (being under-responsive to sensory stimuli, such as not noticing, or taking longer to notice, sounds) and sensory seeking (craving or being fascinated by sensory stimuli, such as seeking out objects that spin) (American Psychiatric Association, 2013).

Sensory reactivity patterns have been found to impact on a wide range of skills associated with daily living, including eating, sleeping, toileting, self-care and hygiene, socialising, fine and gross motor skills, and communication (Bartolomé-Villar et al., 2016; Beaudry-Bellefeuille et al., 2020; Boterberg & Warreyn, 2016; Cermak et al., 2010; Cupelli et al., 2014; Dellapiazza et al., 2018; Glod et al., 2015; Hilton et al., 2007; Hollway et al., 2013; Jasmin et al., 2009; Lane et al., 2010; Mazurek & Petroski, 2015; Paterson & Peck, 2011; Stein et al., 2011; Thye et al., 2018; Watson et al., 2011). Sensory reactivity has further been implicated in mental health concerns in older autistic children, adolescents and adults, with research exploring links between sensory reactivity and anxiety, schizophrenia, depression, bipolar disorder, and obsessive–compulsive disorder (Bailliard, 2015; Bailliard & Whigham, 2017; Engel-Yeger et al., 2016; Glod et al., 2015; Javitt & Freedman, 2015; MacLennan et al., 2020; Rossow et al., 2021; Serafini et al., 2017). More recently, these associations have been shown to extend to mental health symptoms seen in young autistic children as well (MacLennan et al., 2020; Rossow et al., 2021). However, as highlighted in a review of the National Institute of Mental Health’s (NIMH) Research Domain Criteria (RDoC) framework (Harrison et al., 2019), there is a continued need to explore the association between sensory reactivity and mental health in autistic individuals in order to further understand the role sensory reactivity has on the development of mental health symptoms and guide more targeted and effective individualised interventions.

Whilst the most studied mental health condition to be associated with sensory reactivity is anxiety, much less is known about the relationship with other disorders, including depression. Whereas recent research has elucidated specific anxiety subtype relationships with sensory reactivity, and developed a model for understanding the sensory-anxiety relationship (MacLennan et al., 2020), the same degree of enquiry has not yet been extended to depression. Broadly, studies exploring sensory reactivity and depression have been confined to cross-sectional data showing correlations between depression and sensory constructs (Ben-Sasson et al., 2008; Feldman et al., 2020; Pfeiffer et al., 2005). Ben-Sasson et al. (2008) identified an association between both hyper- and hypo-reactivity and depressive symptoms in their sample of autistic children. Pfeiffer and colleagues (Pfeiffer et al., 2005) similarly found an association between hypo-reactivity and depressive symptoms in their autistic adolescent sample. More recently, correlations were found between all three sensory constructs and depressive symptoms; however, the results also identified hyper-reactivity as the only construct that explained a significant proportion of depressive symptom variance (Feldman et al., 2020). In a most recent study of autistic pre-schoolers, hyper-reactivity and sensory seeking were both associated with depressive symptoms (Rossow et al., 2021). However, in that study the relationship was found to be driven by participants who use few to no words.

More broadly, there has been an analogous relationship found between depression and hyper-reactivity in adult studies utilising neurotypical participants (Liss et al., 2008; Serafini et al., 2017), suggesting that the hyper-reactivity-depression relationship may be independent of neurodevelopment. It is posited that factors which underlie sensory reactivity may interact with the factors that contribute to depression, increasing the susceptibility to developing a depressive disorder. More specifically, given the breadth of depressive symptoms—behavioural (self-injurious behaviours), emotional (dysthymia, worthlessness, hopelessness), physiological (reduced or increased appetite, psychomotor agitation or retardation, fatigue, insomnia or hypersomnia) and cognitive (suicidal ideation, impairments in concentration)—it is conceivable that sensory reactivity may directly influence depressive symptoms, with sensory reactivity having previously been implicated in functional impacts on eating (Cermak et al., 2010; Paterson & Peck, 2011; Zobel-Lachiusa et al., 2015), sleep (Hollway et al., 2013; Mazurek & Petroski, 2015; Reynolds et al., 2012) and self-injurious behaviours (Duerden et al., 2012; Malhi & Sankhyan, 2021; Summers et al., 2017) among others.

Whilst the literature reveals sensory reactivity is associated with depressive symptoms, the nature of current published research makes it difficult to interpret directionality or causality. Longitudinal sensory research has previously been conducted with anxiety (Green et al., 2012), however without a similar study design for depressive symptoms, our understanding of causality will remain conjectured. Another critique often cited in the literature on sensory reactivity and mental health, and more broadly noted in general autism and depression research, is the relative lack of research involving participants across the autism spectrum. More specifically, autistic individuals who are pre-verbal, non-verbal or minimally verbal, referred hereafter as those who speak ‘few to no words’ in line with the Module 1 classification algorithm in the Autism Diagnostic Observation Schedule (ADOS-2) (Lord et al., 2012) and endorsed by autism research charities such as Autistica, are often excluded from research studies (Jack & Pelphrey, 2017; Stedman et al., 2019; Tager-Flusberg & Kasari, 2013). Depression research which have included these participants have generally found reduced symptom severity scores in participants who speak few to no words when compared with their verbal peers (Fok & Bal, 2019; Lerner et al., 2018; Skwerer et al., 2019), or lower proportions of participants meeting clinical cut-off for symptom severity (Rossow et al., 2021). However, as described above, more recent research has revealed that the relationship between internalising mental health symptoms, particularly depression, and sensory reactivity in young autistic children appears to be driven by those who speak few to no words (Rossow et al., 2021).

Currently, there are few studies which have explored depressive symptoms or sensory reactivity in autistic children who use few to no words. Further, to the author’s knowledge there is no published research that has yet investigated the predictive relationship between sensory reactivity and depression. As such, the present study seeks to elucidate the predictive relationship between sensory reactivity and depressive symptoms in young autistic children who speak few to no words. In light of previous research, we hypothesize that hyper-reactivity is most likely to be predictively related to depressive symptoms.

## Methods

### Participants

Overall, 33 autistic children (demographic characteristics can be found in Table 1) who used five words or less were recruited through the University of Reading’s Centre for Autism participant database, social media platforms, local National Health Service (NHS) Child and Adolescent Mental Health Services (CAMHS), and through Autism Berkshire—a local autism organisation. Inclusion criteria were (a) either having a standing clinical diagnosis of autism completed by a clinical psychologist and/or speech and language therapist and confirmed by a paediatrician, or having a suspected autism spectrum condition and meeting cut-off criteria on both the Autism Diagnostic Observation Schedule (ADOS-2) (Lord et al., 2012) and the Autism Quotient (AQ) (Auyeung et al., 2008), and (b) having completed the ADOS-2 Module 1 as part of the above assessments. Thirty-two participants completed the ADOS-2 Module 1 to confirm inclusion criteria, and one participant had received a community diagnosis of autism, which included the ADOS-2 Module 1, within the 6 months prior to testing. All participants completed testing at Timepoint 1, with 19 participants also completing testing at the 12 month follow up Timepoint 2. Attempts were further made to contextualise the sample through the use of the Matrix Reasoning subtests of the Wechsler Nonverbal Scale of Ability (WNV) (Wechsler, 2006) for those aged 4 or 5, or the British Ability Scale (BAS3) (Eliot & Smith, 2011) for those aged 3. Five participants were able to complete cognitive testing, with a mean *T*-score of 52.20 (range 33–64, *SD* = 12.80). However, due to the small number of participants who were able to complete this testing, this score may be an over- or under-estimation of the true mean, although caution should be taken if underestimating the intelligence of autistic individuals with few to no words (Courchesne et al., 2019; Giofrè et al., 2019).

**Table 1:** Demographic characteristics of participants

Participants	*N*	*%*	Mean age (Y:M)*	SD (Y:M)*	Range
T1 Total	33		3.91 (3:10)	0.72 (0:8)	3–5
Female	8	24.2	3.75 (3:9)	0.46 (0:5)	3–5
Male	25	75.8	3.96 (3:11)	0.79 (0:9)	3–5
T2 Total	19		4.82 (4:9)	0.66 (0:7)	4–6
Female	4	21.05	4.80 (4:9)	0.45 (0:5)	4–5
Male	15	78.95	4.82 (4:9)	0.73 (0:8)	4–6

Ethnicity	*N*	*%*
Caucasian	16	48.5
South Asian	10	30.3
North African	1	3
Mixed	5	15.2

T2 COVD-19	*N*	Range	Mean	SD
Days in lockdown	19	14–182	59.00	41.04
Had isolated	15			
Days isolated		14–75	35.75	28.90
Impact on child	19	2–4	3.42	.62

Ethics for this study were obtained from both the University of Reading’s Research Ethics Committee and the National Health Service Health Research Authority. Parents provided written consent at Timepoint 1, with voluntary task compliance considered assent in participating children at both testing sessions. All participants completed an in-person testing session at the University of Reading at Timepoint 1. Ten participants completed an in-person testing session at the University of Reading at Timepoint 2, with the remaining participants completing the measures online due to COVID-19 restrictions. None of the participants reported any sight or hearing impairments that would impact their performance in the study.

### Depression Measure

#### Behaviour Assessment System for Children Third Edition: Parent Rating Scale (BASC-3; Preschool and Child versions)

The BASC-3 preschool and child versions are parent-completed measures of adaptive and maladaptive behaviours in children aged 2 to 5 (preschool) and 6 to 11 (child). The measures contain 12 subscales, four composite scales, nine content scales and four executive functioning indices (Reynolds & Kamphaus, 2015), with each item utilising a 4-point Likert-type scale (“Never” to “Almost Always”). This study used the depression subscale, which has 11 questions. Example questions include “cries easily,” “changes mood quickly,” and “is irritable.” Raw scores are summed and transformed into standardised *T*-scores (*M* = 50; *SD* = 10) for interpretation. The BASC-3 preschool and child versions have been found to have both strong internal consistency (Preschool Cronbach’s α = 0.83–0.93; Child Cronbach’s α = 0.86–0.96 across composites and scales) and test–retest reliability (Preschool *r* = 0.87–0.92; Child *r* = 0.85–0.89 across composites and scales) (Reynolds & Kamphaus, 2015). The BASC has been widely used in autism research, including in autistic toddlers and children with a developmental language delay and learning disability (Bradstreet et al., 2017; Ellison et al., 2019; Gardner et al., 2018; Lindsey et al., 2020; Mahan & Matson, 2011). Further, the depression subscale has a reduced reliance on verbal communication, with only one item out of eleven relying on a verbal response (“Says, ‘Nobody likes me’”).

### Sensory Reactivity Measure

#### Sensory Processing Scale Inventory (SPSI)

The SPSI is a parent-report measure of sensory hyper-reactivity, hypo-reactivity and sensory seeking (Schoen et al., 2017), with sensory behavioural items matched to each construct. Example items for hyper-reactivity include “Some background noises bother my child (e.g., humming lights, people talking or music playing in the background);” for hypo-reactivity include “My child does not respond, or is slower to respond, to touch experiences (e.g. leaves clothing twisted on body, does not notice dirty clothes or messy hands);” and for sensory seeking include “My child cannot stop watching visual stimuli, especially contrasting or moving objects (e.g. spinning objects like wheels, fast-changing images on screens, rotating fans).” Items are rated on a binary scoring system (applicable = 1; not applicable = 0), then summed to provide total construct scores. Internal consistency was found to be strong across all scales (SOR: α = 0.89; SUR: α = 0.88; SC: α = 0.93) (Schoen et al., 2017).

### Additional COVID-19 Questions

In order to contextualise the impact of the COVID-19 pandemic, participants were also asked to report how many days they had been in local or national lockdown for at the time of their second assessment session, how long they had had to self-isolate if showing symptoms of, or being in close contact with some showing symptoms of, COVID-19, and how much parents felt the lockdown restrictions had impacted on their child (rated on a 4-point Likert-type scale; 1 = not at all, 2 = a small amount, 3 = a moderate amount, 4 = an extensive amount) (see Table 1).

### Analysis

SPSS 24 was used to analyse the data. As the analyses were exploratory, the study was not pre-registered. A Shapiro–Wilk test indicated all variables met assumptions of normality. Pearson correlation analysis was used to elucidate the correlational relationships between hyper-reactivity, hypo-reactivity, sensory seeking and depressive symptoms at Timepoint 1 and Timepoint 2. Pearson correlation analyses was also used to understand the relationship between hyper-reactivity, hypo-reactivity, sensory seeking and depressive symptoms between timepoints. Following this we conducted a series of hierarchical linear regressions to understand the predictive relationship between hyper-reactivity, hypo-reactivity, sensory seeking and depressive symptoms across timepoints.

### Community Involvement

The design of this study, and the research questions developed, were aligned with Autistica’s research priorities, which were produced with support from stakeholders. Throughout our project we engaged the families who took part, and sought feedback on our testing sessions and procedures, interpretations of our results and the language used in the article.

## Results

### Descriptive Analysis

Frequencies of participants meeting clinical threshold depressive symptoms were assessed. As per Reynolds and Kamphaus (2015), clinically indicated scores are determined as a T-score of 70 or higher in the domains assessed, representing less than 5% of the population. 18.2% of our sample scored in the clinically significant range for depressive symptoms at Timepoint 1 (T-score range 37–79, *M* = 59.31, *SD* = 11.45), and 12.1% scored in the clinically significant range for depressive symptoms at Timepoint 2 (T-score range 43–86, *M* = 62.05, *SD* = 12.39).

### Correlation Analyses

Given the COVID-19 pandemic and associated lockdowns, we first assessed if lockdown, self-isolation or impact of the pandemic were related to sensory reactivity or depressive symptoms at Timepoint 2. Bivariate correlational analyses were conducted between sensory reactivity and depression, and pandemic factors (number of days in lockdown, number of days self-isolating, and parents’ perception of pandemic impact on their child). There were no significant effects detected (*p* > 0.05) (see Appendix A).

In order to examine the relationship between sensory reactivity and depression symptoms in our sample across both timepoints, bivariate correlational analyses were conducted between the SPSI sensory construct total scores and BASC-3 depression scores (Table 2).

**Table 2:** Correlation matrix between raw scores of the SPSI and the BASC-3 depression subscale

	1	2	3	4	5	6	7
**Timepoint 1**							
1. Hyper-reactivity							
2. Hypo-reactivity	.44^*^						
3. Sensory Seeking	.39^*^	.31					
4. Depression	.67^***^	.07	.51^**^				
**Timepoint 2**							
5. Hyper-reactivity	.57^**^	.38	.30	.58^**^			
6. Hypo-reactivity	.44^*^	.74^***^	.25	.12	.31		
7. Sensory seeking	.40	.45^*^	.80^***^	.62^**^	.60^**^	.41	
8. Depression	.56^*^	.26	.72^**^	.76^***^	.67^**^	.23	.70^**^

*SPSI* Sensory Processing Scale Inventory, *BASC-3* Behaviour Assessment System for Children, 3rd Edition

*p < .05; **p < .01; ***p > .001

#### Timepoint 1

Moderate positive effects were detected between depression and hyper-reactivity (r = 0.67, 95% CI [0.395, 0.829], *p* < 0.001), and depression and sensory seeking (r = 0.51, 95% CI [0.173, 0.737], *p* = 0.005).

#### Timepoint 2

Moderate to large positive effects were detected between depression and hyper-reactivity (r = 0.67, 95% CI [0.304, 0.860], *p* = 0.002), and depression and sensory seeking (r = 0.70, 95% CI [0.348, 0.880], *p* = 0.001).

#### Timepoint 1 (T1) and Timepoint 2 (T2)

Moderate to large positive effects were detected between T1 depression and T2 hyper-reactivity (r = 0.58, 95% CI [0.181, 0.812, *p* = 0.008), T2 sensory seeking (r = 0.62, 95% CI [0.226, 0.837, *p* = 0.005), and T2 depression (r = 0.76, 95% CI [0.458, 0.901], *p* < 0.001). Moderate positive effects were detected between T1 hyper-reactivity and T2 hyper-reactivity (r = 0.57, 95% CI [0.185, 0.804], *p* = 0.007), T2 hypo-reactivity (r = 0.44, 95% CI [0.007, 0.731], *p* = 0.048), and T2 sensory seeking (r = 0.56, 95% CI [0.135, 0.806], *p* = 0.014). Moderate to large positive effects were detected between T1 hypo-reactivity and T2 hypo-reactivity (r = 0.74, 95% CI [0.457, 0.889], *p* < 0.001) and T2 sensory seeking (r = 0.45, 95% CI [0.004, 0.742], *p* = 0.049). Large positive effects were detected between T1 sensory seeking and T2 sensory seeking (r = 0.80, 95% CI [0.552, 0.917], *p* < 0.001) and T2 depression (r = 0.72, 95% CI [0.392, 0.884], *p* < 0.001).

### Regression Analyses

**Fig. 1:**
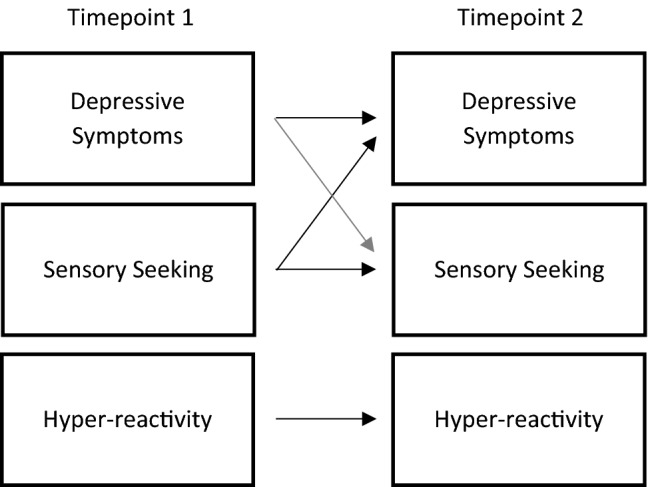
Basic model of predictive relationship between Timepoint 1 and Timepoint 2 depressive symptoms, sensory seeking and hyper-reactivity in autistic children who speak few to no words

Three hierarchical linear regressions were conducted to ascertain the predictive relationship between sensory constructs and depression. For the below regressions, hypo-reactivity was not included as no relationship was found to depression at either T1 or T2. In line with previous research, the control variable was entered in Step 1 (for example, when looking at the predictive relationship of T1 depression on Timepoint 2 variables, T2 depression was entered as the control variable in Step 1), with subsequent variables entered descending according to strength of correlation (Sáez-Suanes et al., 2020). The regression analyses overview can be found in Table 3 and a model of the basic predictive relationship can be found in Fig. 1.

**Table 3:** Hierarchical regression analysis summary for depression, sensory seeking and hyper-reactivity

	Step 1 (control)			Step 2			Step 3		
Variable	*B*	*SE*	β	*B*	*SE*	β	*B*	*SE*	β
**Depression (T2)**									
Depression (T1)	.784	.165	.755***	.534	.173	.514**	.497	.203	.479*
Sensory seeking (T1)				.544	.211	.429*	.529	.221	.418*
Hyper-reactivity (T1)							.067	.178	.068
*R*^*2*^		.571			.697			.699	
Adjusted *R*^*2*^		.545			.659			.639	
*R*^*2*^ change					.126			.003	
*F* for change in *R*^*2*^					6.648*			.140	
**Sensory seeking (T2)**									
Sensory Seeking (T1)	.919	.158	.816***	.761	.156	.675***	.777	.161	.690***
Depression (T1)				.328	.139	.326*	.379	.167	.377*
Hyper-reactivity (T1)							-.079	.135	-.095
*R*^*2*^		.665			.751			.757	
Adjusted *R*^*2*^		.645			.720			.708	
*R*^*2*^ Change					.086			.006	
*F* for change in *R*^*2*^					5.553*			.343	
**Hyper-reactivity (T2)**									
Hyper-reactivity (T1)	.562	.200	.552*	.322	.245	.316	.314	.255	.309
Depression (T1)				.411	.260	.381	.391	.288	.362
Sensory seeking (T1)							.059	.305	.044
*R*^*2*^		.305			.394			.396	
Adjusted *R*^*2*^		.267			.323			.282	
*R*^*2*^ Change					.089			.001	
*F* for change in *R*^*2*^					2.502			.037	

*T1* Timepoint 1, *T2* Timepoint 2

*p < .05; **p < .01; ***p > .001

#### Depression

Depression at T1 explained 57% of the variance in T2 depression (β = 0.76, *p* < 0.001). The addition of T1 sensory seeking significantly improved the prediction of T2 depression (ΔR^2^ = 13%, F_change_ = 6.65, *p* = 0.020). The addition of T1 hyper-reactivity did not significantly improve the prediction of T2 depression (ΔR^2^ < 1%, F_change_ = 0.140, *p* = 0.714).

#### Sensory Seeking

Sensory seeking at T1 explained 67% of the variance in T2 sensory seeking (β = 0.82, *p* < 0.001). The addition of T1 depression significantly improved the prediction of T2 sensory seeking (ΔR^2^ = 9%, F_change_ = 5.55, *p* = 0.032). The addition of T1 hyper-reactivity did not significantly improve the prediction of T2 depression (ΔR^2^ < 1%, F_change_ = 0.343, *p* = 0.567).

#### Hyper-reactivity

Hyper-reactivity at T1 explained 31% of the variance in T2 hyper-reactivity (β = 0.55, *p* = 0.012). The addition of T1 depression did not significantly improve the prediction of T2 hyper-reactivity (ΔR^2^ = 9%, F_change_ = 2.502, *p* = 0.132), nor did the addition of sensory seeking (ΔR^2^ < 1%, F_change_ = 0.037, *p* = 0.850).

## Discussion

The objective of this study was to elucidate the predictive relationship between sensory reactivity and depressive symptoms in young autistic children who speak few to no words. Our findings show a relationship between hyper-reactivity and sensory seeking and depressive symptoms, and are the first to suggest that there is a bidirectional predictive relationship between sensory seeking and depressive symptoms over time.

Depressive symptoms were associated with sensory hyper-reactivity and sensory seeking at both timepoints. As depressive symptoms increased, so too did sensory reactivity. This is consistent with previous research with autistic children which have found a similar relationship between hyper-reactivity and depressive symptoms (Ben-Sasson et al., 2008; Bitsika et al., 2016; Pfeiffer et al., 2005) and sensory seeking and depressive symptoms (Bitsika et al., 2016; Rossow et al., 2021). However, our data differs from previous research in that we did not find a relationship between depressive symptoms and hypo-reactivity (Bitsika et al., 2016; Pfeiffer et al., 2005). Differences in previous findings and our findings may be due to differences in sample and methodology. Our study utilised a younger sample whilst previous studies used older samples, with mean ages of 11.2 years (Bitsika et al., 2016) and 9.8 years (Pfeiffer et al., 2005). Further, our sample exclusively included participants who use few to no words, whereas previous studies included verbal participants.

Unsurprisingly, baseline sensory and depressive symptoms predicted later sensory and depressive symptoms respectfully. However, the data further show a bidirectional predictive relationship between sensory seeking and depression. Young autistic children who showed sensory seeking at baseline later developed higher levels of depressive symptoms, and those who had baseline depressive symptoms developed greater sensory seeking behaviours. It is posited that there may be a cyclical relationship whereby seeking sensory input without efficient and effective integration contributes to the development of depressive symptoms, which in turn drives further sensory seeking as a form of coping strategy to support physiological, behavioural and emotional regulation. The sensory-depression relationship therefore appears to be more complex than just a linear development of depressive symptoms in response to sensory discomfort, which further helps explain the lack of a predictive association between depressive symptoms and hyper-reactivity. An alternative hypothesis is that, in a parent-reported context, sensory seeking may be erroneously endorsed as depressive symptoms, such as social withdrawal due to a focus on a sensory stimuli or mood changes due to a preferred sensory activity being interrupted. Further exploration of this is needed to ascertain the underlying mechanisms.

In considering previous research citing the relationship between sensory hyper- and hypo-reactivity and depression (Ben-Sasson et al., 2008; Pfeiffer et al., 2005), it could be suggested that for those who speak few to no words and seek sensory input when young, but whose needs are consistently not met over time, develop a sense of defencelessness or powerlessness to modulating such sensory inputs. Consequently, they may become despondent and cease reacting to sensory stimuli (hypo-reactive) as they become older, akin to learned helplessness (Abramson et al., 1978). This may be a possible starting point in explaining the association with hypo-reactivity in older autistic children and adolescents. Further exploration of this hypothesis is needed, particularly as there is conjecture over the general stability of sensory reactivity over time (Baranek et al., 2019; McCormick et al., 2016; Perez Repetto et al., 2017; Wolff et al., 2019). Another possibility is the misattribution of depressive symptoms as hypo-reactivity. Previous studies utilised the Sensory Profile (Dunn, 1999), which uses items for hypo-reactivity including “does things in a harder way than is needed (for example, wastes time, moves slowly)” and “seems to have low self-esteem (for example, difficulty liking self).” The inclusion of explicit emotion-based items may obfuscate the true hypo-reactivity-depression relationship. A further explanation is that the difference seen may also simply be a consequence of sample characteristics, in that our sample included younger autistic participants who had few to no words and use limited verbal language, whereas previous studies used older and verbal samples (Bitsika et al., 2016; Pfeiffer et al., 2005). There is evidence to show that in autistic people the development of depression increases with age, particularly in adolescence and early adulthood (e.g. Mayes et al., 2011; Uljarević et al., 2020). As such, and given sensory reactivity may also not be stable longitudinally, the relationship to sensory reactivity may also change over time and reflect the discrepancy between findings. This will remain speculative without further exploration utilising older children, adolescents and adults who use few to no words.

This study was unique in that it explored the longitudinal relationship between sensory reactivity and depressive symptoms in young autistic children who use few to no words. However, there are several limitations noted. Firstly, this study only analysed data from parent-report measures, and was not able to incorporate data from other sources, such as observational measures, as previous research has recommended (Siper et al., 2017; Tavassoli et al., 2016, 2019). The BASC-3 was used in this study as there are few standardised measures of depressive symptoms for young children; however, it also highlights a reliance, particularly in autism and child research, on parent-report measures to the detriment of measures of observation, physiology and self-report. Whilst attempts were made and observational data was collected at Timepoint 1 using the Sensory Assessment of Neurodevelopmental Differences (SAND) (Siper et al., 2017), the COVID-19 pandemic hindered the collection of observational data at Timepoint 2 and as such there was insufficient data to analyse. The use of a parent-report measure of depressive symptoms in the current study may increase the risk of a bias towards the over- or under-responding of mental health symptoms, the misattribution of autistic and sensory behaviours or the defining of behaviours through a non-autistic viewpoint. For example, participants who use few to no words may have items incorrectly ascribed where the underlying factor may be unrelated to depression or sensory reactivity, such as pain from a comorbid health need, temper-outbursts linked to executive dysfunction, or frustration due to communication difficulties. Future research should aim to develop and utilise measures of mental health which have been specifically designed and validated with and for autistic people, particularly with autistic people who use few to no words. The authors acknowledge that work to validate a measure of depression for use with autistic adults is currently under development (Cassidy et al., 2021).

Further, whilst our sample size was congruent with previous studies of sensory reactivity and mental health (Leekam et al., 2007; Pfeiffer et al., 2005; Rossow et al., 2021) and acceptable for the analyses we conducted (Jenkins & Quintana-Ascencio, 2020), sample size, attrition and data limitations meant we were not able to explore the potential impact of demographic factors such as gender or socioeconomic differences. Recent research has indicated that autistic women and non-binary people experience depression at higher rates than autistic men (Sedgewick et al., 2021), and there may also be gender differences in sensory reactivity as well (Walsh et al., 2018). Future research exploring the potential impact of cultural and demographic differences, such as gender, is needed to clarify whether such factors impact on the sensory-depression association. Additionally, we were unable to utilise other methods, such as structural equation modelling, to further explore the predictive relationships between sensory reactivity and depressive symptoms.

Our findings have implications for interventions with young autistic children who speak few to no words. Sensory seeking is related to the development of depressive symptoms, with evidence suggesting a complex relationship whereby sensory seeking both influences the development of, and is used as a coping mechanism to reduce, depressive symptoms. Interventions targeting mood in young autistic children should also consider supporting their hyper-reactivity and sensory seeking behaviours. This should not only include direct sensory and behavioural approaches, such as prescribed sensory strategies (Wilbarger, 1995) or environmental adjustments that reduce the likelihood of inducing stimming (Kapp et al., 2019), but also support for the development of functional communication (for example key word signing or other augmented communication tools). Consideration of sensory differences, particularly for those who present with cooccurring depressive symptoms, may help in the development of more robust support plans and interventions.

Further exploration of the link between sensory reactivity and depression is required, as too is the development of validated measures for depression in autistic children. Additionally, it is fundamental that future research endeavour to include participants who present across the autism spectrum, and particularly those who speak few to no words. It is also critical to continue longitudinal research to consolidate the sensory-mood causal relationship, especially in older children, adolescents and adults. It is also worth considering a clinical exploration of sensory interventions in the treatment or prevention of depressive symptoms. This would provide a clearer understanding of whether addressing sensory needs in autistic young people is helpful in the long-term prevention of depressive symptoms. Further, future research could also explore additional variable relationships which could not be assessed in the current study, such as in sensory domains (e.g. auditory, tactile, visual, interoceptive domains), item- level analyses, or demographic factors such as gender. Studies with larger sample sizes are also required to replicate and fully understand the sensory-depression relationship.

## Conclusion

The objectives of this study were to explore the predictive relationship between sensory reactivity and depressive symptoms in young autistic children who speak few to no words. Sensory hyper-reactivity and seeking were found to be related to depressive symptoms, and there appears to be a bidirectional predictive relationship between sensory seeking and depressive symptoms. These findings have important implications for preventative and early interventions for depression in autistic children.

## Supplementary Information

Below is the link to the electronic supplementary material.

Supplementary file1 (DOCX 18 kb)
