# Supplementary material for: The Predictive Relationship Between Sensory Reactivity and Depressive Symptoms in Young Autistic Children with Few to No Words
Source: J Autism Dev Disord. 2022 Mar 26;53(6):2384–94. doi: 10.1007/s10803-022-05528-9 (PMC8956453; doi:10.1007/s10803-022-05528-9)
Supplement: Supplementary file 1 — Supplementary file1 (DOCX 18 kb) [file 10803_2022_5528_MOESM1_ESM.docx]

| **Appendix A**  *Correlation matrix between Timepoint 2 raw scores of the SPSI, BASC-3 depression subscale and COVID-9 pandemic factors.* | | | | | | | |
| --- | --- | --- | --- | --- | --- | --- | --- |
|  | Days in lockdown | | Days isolating | | Impact on child | |  |
|  | *r* | *p* | *r* | *p* | *r* | *p* |  |
| Hyper-reactivity | .277 | .317 | .173 | .480 | .010 | .967 |  |
| Hypo-reactivity | -.066 | .814 | -.410 | .081 | -.218 | .369 |  |
| Sensory Seeking | .263 | .343 | -.222 | .360 | .019 | .938 |  |
| Depression | .201 | .509 | -.233 | .367 | .182 | .485 |  |
| **Note**: SPSI = Sensory Processing Scale Inventory; BASC-3 = Behaviour Assessment System for Children, 3rd Edition. | | | | | | |  |
